# Editorial for Special Issue “Interactions between Plant Beneficial *Pseudomonas* spp. and Their Host”

**DOI:** 10.3390/microorganisms11102591

**Published:** 2023-10-20

**Authors:** Martin Filion

**Affiliations:** Science and Technology Branch, Saint-Jean-sur-Richelieu Research and Development Centre, Agriculture and Agri-Food Canada, Government of Canada, 430 Gouin Boul., Saint-Jean-sur-Richelieu, QC J3B 3E6, Canada; martin.filion@agr.gc.ca

Plant-beneficial *Pseudomonas* spp. are aerobic, Gram-negative bacteria that are well known for their great metabolic flexibility and lifestyle adaptability, allowing them to colonize a wide range of environmental niches, including plant roots and their associated soil (the rhizosphere), as well as plant aerial surfaces (the phyllosphere). Many strains of plant-beneficial *Pseudomonas* spp. display plant growth-promoting and/or biocontrol activity against various plant pathogens. Some strains promote plant growth by rendering phosphorus, nitrogen and iron more available, as well as producing beneficial phytohormones such as indole-3-acetic acid. Others instead display biocontrol capabilities by actively competing with other microorganisms, stimulating the plant immune system, and producing several antimicrobial molecules with antagonistic effects against phytopathogens. The ability of plant-beneficial *Pseudomonas* spp. to metabolize a wide array of nutrients, their rapidity and ease of growth, and their natural abundance in a variety of plant-soil environments make them promising organisms for the development of commercial biofertilizer and biocontrol products.

This Special Issue of *Microorganisms* gathers 8 articles addressing various aspects related to the ecology [1,2,3,4], diversity [5], physiology [6] and genetics [1,4,5,6,7] of plant-beneficial *Pseudomonas* spp., while putting special emphasis on the mechanisms involved in biocontrol [1,2,4,5,8] and/or plant growth promotion [3,6,8]. The articles cover research conducted using different plant hosts, experimental systems and conditions. Altogether, we think that this Special Issue provides a valuable update on important aspects related to the interactions occurring between plant beneficial *Pseudomonas* spp. and their host.
